# Does the cost function of human motor control depend on the internal metabolic state?

**DOI:** 10.1186/1471-2202-12-S1-P99

**Published:** 2011-07-18

**Authors:** Scott V Taylor, Aldo A Faisal

**Affiliations:** 1 Department of Bioengineering, Imperial College London, London, SW7 2AZ, UK; 2 Department of Computing, Imperial College London, London, SW7 2AZ, UK

## 

Optimal feedback control theory (OFCT) has been very successful in explaining human motor coordination in a principled manner [1,2]. OFCT derives motor control policies from the minimization of a cost function, which predicts a large variety of movement data [3]. However, very little is known about the nature of this cost function. From the many proposed costs (which take the same mathematical form) two stand out as being motivated in a principled manner: noise [4,5] and “effort” (incl. muscle fatigue and metabolic demand [6]). These two cost are evolutionarily sensible as they maximize an organism’s fitness: increasing task-relevant precision and energy efficiency. It was recently suggested [6] that noise and effort are weighed against each other when determining motor coordination. However, the neuronal implementation or representation of such costs in the brain remains unclear [7]. We test the hypothesis that this trade-off may be directly affected by our internal metabolic state (e.g. blood glucose level). We test the hypothesis if a subject’s internal metabolic state has an impact on the strategy for motor control. Specifically, low metabolic levels could bias motor coordination across redundant muscle groups from larger, metabolically more costly muscles towards smaller muscles to increase efficiency.

We performed preliminary experiments by conducting reaching experiments under a dietary regime. 5 right-handed participants 21-27 years old performed reaching movements in a virtual reality arm movement-tracking rig (visual stimuli were projected via a mirror system onto the plane of hand movement). Air sleds on a surrounding table supported the participant’s arm to allow frictionless movement. The task began when participants moved their hand to a visual workspace centre; then a 1.5cm radius target sphere appeared 15cm away in one of 8 directions (total of 800 trials, randomly ordered directions). Subjects chose when to start reaching towards the target, having to come to a stop within the target sphere within movement durations of 75 to 125ms. Feedback was given in the form of a score that increased for successful trials and decreased conversely. Each experiment involved two morning sessions on separate days. 3 subjects followed their normal eating/drinking routine for the first session then fasted from 8.00pm the evening before the second session and vice versa for the 2 other subjects. Blood glucose measurements were taken before and after each session using a personal blood glucose monitoring system.

Our preliminary data suggests a systematic shift in the coordination of muscle groups acting on each joint based on available energy levels. We found statistically significant changes in shoulder and elbow joint utilisation (integrated absolute change in joint angle) in all 5 subjects and in up to 5 out of 8 target directions depending on the metabolic state. Moreover, we can model the subject’s reaching trajectories under both metabolic conditions using OFCT by a change in the weight of the metabolic cost term as a function of internal metabolic state. Our preliminary findings are important as we link for the first time metabolic state to neuronal computations underlying motor control.
